# Physiotherapy for Prevention and Treatment of Fecal Incontinence in Women—Systematic Review of Methods

**DOI:** 10.3390/jcm9103255

**Published:** 2020-10-12

**Authors:** Agnieszka Irena Mazur-Bialy, Daria Kołomańska-Bogucka, Marcin Opławski, Sabina Tim

**Affiliations:** 1 Department of Biomechanics and Kinesiology, Faculty of Health Science, Jagiellonian University Medical College, Grzegorzecka 20, 31-531 Krakow, Poland; daria.kolomanska@gmail.com (D.K.-B.); sabina.tim@doctoral.uj.edu.pl (S.T.); 2 Department of Gynecology and Obstetrics with Gynecologic Oncology, Ludwik Rydygier Memorial Specialized Hospital, Zlotej Jesieni 1, 31-826 Kraków, Poland; oplawski.m@gmail.com

**Keywords:** fecal incontinence, physiotherapy, pelvic floor rehabilitation, biofeedback, electrostimulation

## Abstract

Fecal incontinence (FI) affects approximately 0.25–6% of the population, both men and women. The most common causes of FI are damage to/weakness of the anal sphincter muscle and/or pelvic floor muscles, as well as neurological changes in the central or peripheral nervous system. The purpose of this study is to report the results of a systematic review of the possibilities and effectiveness of physiotherapy techniques for the prevention and treatment of FI in women. For this purpose, the PubMed, Embase, and Web of Science databases were searched for 2000–2020. A total of 22 publications qualified for detailed analysis. The studies showed that biofeedback (BF), anal sphincter muscle exercises, pelvic floor muscle training (PFMT), and electrostimulation (ES) are effective in relieving FI symptoms, as reflected in the International Continence Society recommendations (BF: level A; PFMT and ES: level B). Research has confirmed that physiotherapy, by improving muscle strength, endurance, and anal sensation, is beneficial in the prevention of FI, both as an independent method of conservative treatment or in pre/post-surgery treatment. Moreover, it can significantly improve the quality of life of patients. In conclusion, physiotherapy (in particular, BF, PFMT, or ES, as effective methods) should be one of the key elements in the comprehensive therapy of patients with FI.

## 1. Introduction

Fecal incontinence (FI) has been defined by the International Continence Society (ICS) as the involuntary loss of liquid and/or solid stool [1]. The intimate nature of the condition makes it difficult to accurately determine its prevalence [2]. It has been estimated that FI affects 0.25–6% of the population [3]. It concerns both men and women. It more often affects the elderly, especially patients staying in nursing homes, and it may be associated with limited mobility and care possibilities [4].

FI can be divided into sub-types: passive FI, when the stool is leaking without any previous feeling of pressure, urge FI (from overfilling), when the patient feels a push but is unable to stand up long enough to go to the toilet, and exercise FI, appearing when intra-abdominal pressure increases [1,5].

The cause of fecal incontinence is the improper functioning of anal sphincter muscles, as a result of their damage and/or neurological changes including the disturbance perception of sensory stimuli from the anal canal. The weakness of anal sphincter muscles may be caused by perinatal trauma (e.g., perineal fractures) or trauma during proctological procedures [6]. FI may be caused by disease entities associated with frequent diarrhea, including irritable bowel syndrome, Crohn’s syndrome, or other bowel diseases [7]. Neurological changes may be caused by trauma, cancer, or degenerative diseases in the central nervous system [8]. FI may also be caused by advanced diabetes mellitus with polyneuropathy, degenerative changes in muscles (atrophy) and nerves (neuropathy) and pelvic floor muscle (PFM) dysfunction [5,6,7,8,9]. Risk factors for FI are age, female gender, obesity, smoking, pharmacotherapy, weakness and trauma of PFM, and the coexistence of urinary incontinence and organ prolapses [10,11]. The causes of FI differ between women and men. Among men, the problem of anorectal sensation disorders is more common. Women, on the other hand, are more often affected by anal sphincter muscles disorders, on the grounds of obstetric trauma and weakened PFM [11,12].

Considering the prevalence of the FI problem, it is important to create a comprehensive system of treatment, part of which should be physiotherapy. Therefore, the aim of this study was to perform a systematic review of the literature on the possibilities and effectiveness of physiotherapeutic techniques, both in the treatment of women with FI as well as for the prevention of the above problem.

### 1.1. Factors Determining Fecal Continence

Fecal continence is possible due to the appropriate integrity of anatomical structures and neuromuscular function of the rectum and PFM, as well as rectal compliance and consistency of stool [13,14]. The anatomical structures responsible for stool continence include the rectum, anal canal, and PFM, including anal sphincters. The rectum provides continence by maintaining lower pressure than in the anal canal. The mucous membrane of the anal canal is filled with internal hemorrhoid tissue which, when expanded, helps to maintain proper resting tonus and ensures tightness [14,15]. The rectum is closed by the permanent tonic action of the internal sphincter muscle and its action can be supported by the voluntary contraction of the external sphincter muscle [16]. The internal sphincter of the anus is a smooth muscle that provides 70% of the natural barrier for fecal continence [17]. In turn, the puborectalis muscle, together with external anal sphincter muscle, are responsible for the conscious maintenance of the stool while sensing pressure before defecation. The above two muscles are striated muscles, mainly composed of slow twitch, type I fibers [18]. The rectum is supported by the levator ani muscle, which belongs to the pelvic floor muscles that support from below the rectum [13]. Part of the levator ani muscle, the puborectalis muscle, moving away from the pubic bone, runs backwards and wraps up in the form of loops around the rectum, forming the so-called Parks angle (i.e., the angle between the anus and the rectum) and closing the anal canal [19]. At rest, the anorectal angle forms a right angle, during conscious muscular contraction it is at 70°, while during defecation, it increases to 110–130° [20].

The anorectum is rich in sensory, motor, and autonomic innervations [19]. The internal anal sphincter and the rectum are controlled by the parasympathetic and sympathetic sacral nerves, while the external anal sphincter muscle is controlled by the vulva nerve [21]. The sympathetic system stimulates muscular contraction (i.e., closure of the rectum), while the parasympathetic system relaxes the muscles, allowing for defecation [22]. The sphincter mechanism reacts differently, depending on the consistency of the stool, as during defecation and anal stretching, the sensory receptors are irritated. In response to anal stretching, the inhibitory reflex is triggered. The internal sphincter is relaxed, preparing the anal canal for defecation [23]. Then, the remaining pelvic floor muscles also relax, and bowel movements occur. The defecation process can be stopped by voluntary contraction of the external anal sphincter and the puborectalis muscle. Rectal contractions and the feeling of urgency on the stool disappears [17].

The problem of fecal incontinence occurs when the rectal pressure is higher than the anal sphincter pressure. When the anal sphincter is weakened or damaged as a result of increased intra-abdominal pressure (e.g., chronic coughing, abnormal lifting of objects, or obesity), uncontrolled leakage of stool may occur [24]. Pelvic muscle contraction stabilizes the pelvic floor organs, in relation to the connective tissue structures. Therefore, damage to PFM or ligaments (e.g., pubourethral or sacrouterine ligament) may disturb the proper stool continence [9]. Damage to one element (e.g., nerve, muscle, or ligaments) may lead to the dysfunction of other components [21]. Nerve dysfunction results in impaired motor control, which may lead to constipation, rectal pain, and fecal incontinence [25]. In the case of decreased rectal sensation, when the rectum is stretched by the stool immediate contraction of the external sphincter is impossible, which leads to the loss of stool. On the other hand, rectal hypersensitivity may lead to disproportionately higher sphincter contraction force compared to rectal filling [17].

### 1.2. Physiotherapeutic Diagnosis in Fecal Incontinence

Physiotherapeutic diagnosis in fecal incontinence includes a detailed history, *per vaginam* and/or *per rectum* palpation, and global postural evaluation [16,26,27]. In the interview, the therapist should consider questions about the onset of symptoms, their frequency, severity, consistency of stool, coexisting diseases, conditions, previous injuries, medications, and diet; for women, additional questions should be asked about their parturitions and course [16]. The pelvis, due to its numerous muscular attachments, is exposed to muscle imbalance and the influence of disorders even in distant parts of the body [27]. Studies have confirmed that disorders of spinal curvature and increased posture defects can affect the development of abnormal respiratory patterns and lead to increased additional abdominal pressure, which negatively affects the PFM [27,28]. The PFM works together with the diaphragm and abdominal muscles during respiration. Therefore, the respiratory pattern should also be assessed [29]. The physiotherapeutic examination should include a global assessment of posture, spinal mobility, diaphragm, and an examination of the length, strength, and tension of the muscles that have attachments on the bones forming the pelvis, as well as pelvic angle and the positions of hips, knees, and feet [27].

Another element of physiotherapeutic diagnosis of PFM is *per vaginam* and/or *per rectum* examination. These allow for the assessment of the correctness and effectiveness of PFM work. The examination includes voluntary (will-dependent) contraction, used during PFM training, and involuntary (will-independent) contraction, occurring during increased intra-abdominal pressure [30,31]. The physiotherapist may also use EMG, perineometer, and palpation to evaluate the PFM function [32,33]. The perineometer is used to assesses vaginal pressure, while the EMG records the electrical activity of muscle fibers [32,33,34]. PFM palpation is a common, fast, and inexpensive examination which should be performed by an experienced therapist. PFM palpation is widely used in both diagnostics and scientific research [32].

The PERFECT system with modified OXFORD scale is used for the *per vaginam* and/or *per rectum* palpation [35]. Laycock et al. described the PERFECT scale as a test for power (P), endurance (E), slow-twitch fiber capacity (R), fast-twitch fiber capacity (F), PFM contraction standard (E), abdominal transverse muscle contraction (C), and PFM involuntary contraction when abdominal pressure increasing during cough (T) [26,34]. The strength of the PFM is assessed using the Modified Oxford Scale, in which 0 to 5 points can be obtained, where 0 means no contraction and 5 means strong contraction against resistance [26,30]. A detailed description of the PERFECT test scheme is given in Table 1. Briefly, in order to assess the PFM strength, the woman is asked to keep the contraction as long as possible. Strength is expressed as the time when the strength of the maximum contraction decreases to half of its value. Examination of the efficiency of the slow twitch fibers is based on the highest possible number of contractions of maximum strength [34]. On the other hand, the efficiency of fast twitch fibers is assessed by the number of maximum one-second contractions and the fastest possible muscle relaxation between contractions [30]. The physiotherapeutic diagnosis of PFM also evaluates the contraction pattern. During voluntary and involuntary contractions, the PFM should lift up in a cefalo-ventral manner [34]. The co-contraction of the transverse abdominal muscle, which should occur together with both arbitrary and involuntary PFM contraction, is assessed further [36]. The reflective reaction of PFM to an increase in intra-abdominal pressure (cough test) is assessed as the last element. The PFM contraction appearing with increased abdominal pressure protects the pelvic floor against pelvic organ depression [32]. The palpation test also pays attention to muscle tonus, where the therapist checks whether the resting pressure of PFM is normal, lowered, or increased [34,37].

A global study of posture, pelvic statics, and pelvic floor muscles, completed with appropriate questionnaires, allows for an accurate functional evaluation of PFM, as well as the proper conduct of therapy [38]. The questionnaires most commonly used in fecal incontinence are the Wexner scale of incontinence severity [39], the Fecal Incontinence Quality of life (FIQOL) [40], the Fecal Incontinence Severity Index (FISI) [41], and Fecal Incontinence Severity Score (FISS) [42].

## 2. Materials and Methods

The literature review was conducted in the Medline-PubMed, Embase, and Web of Science databases. The keywords used were the following expressions: fecal incontinence physiotherapy, fecal incontinence rehabilitation, fecal incontinence physical exercises, fecal incontinence exercises, fecal incontinence biofeedback, fecal incontinence magnetic stimulation, fecal incontinence electrical stimulation, and fecal incontinence pelvic floor training.

The review included studies on the influence of various physiotherapeutic methods in the treatment and prevention of fecal incontinence in women. Articles only in English with studies published from January 2000 to April 2020 were qualified. The exclusion criteria were as follows: Language of publication other than English, conducting or publishing the studies before 2000, and/or failure to demonstrate the influence of physiotherapy on the treatment of fecal incontinence in women. Furthermore, articles in which men were also examined, in which it was impossible to separate the results for women only, were also rejected. Systematic reviews, letters to the editorial office, master’s or doctoral theses, summaries of conference speeches, study protocols, and studies that did not address fecal incontinence in women were not included. The review was conducted by two independent authors with the methodology following the PRISMA (Preferred Reporting Items for Systematic Reviews and Meta-Analyses) rules.

## 3. Results

**Figure 1:**
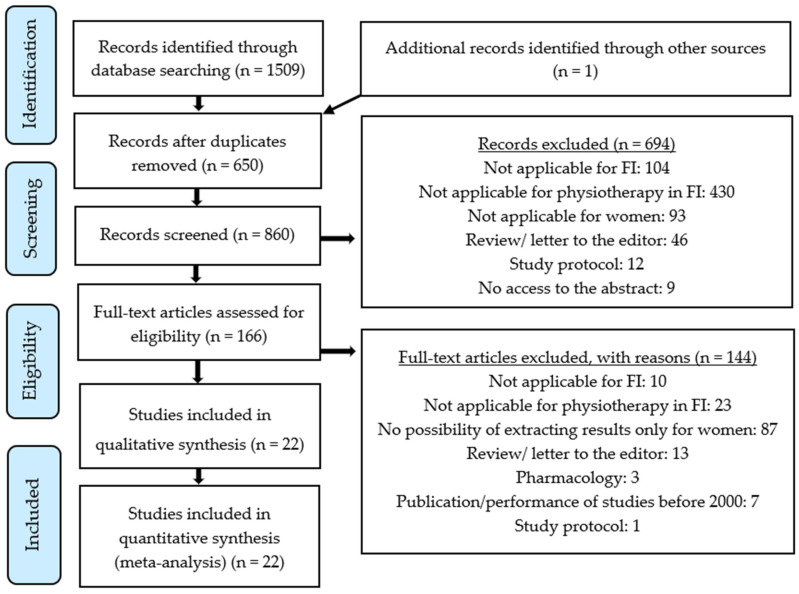
PRISMA diagram presenting the different phases of the systematic review.

In total, 1510 articles were found. After removing duplicates, 860 publications remained. Finally, 166 articles were left to be fully read. On the basis of inclusion and exclusion criteria, 22 publications qualified for review. The PRISMA diagram (Figure 1) was used to describe the particular stages of the review. The diagram presents the reasons for publication exclusions and the final number of studies included in the analysis. Randomized studies were evaluated using the Pedro scale (Table 2). The Pedro scale is used to critically assess randomized clinical trials. Publications are evaluated in 11 categories, with a maximum score of 10 points (eligibility criteria are not included in the total score) [43]. Moreover, Table 3 presents a short description of the qualified studies.

## 4. Prevention of Fecal Incontinence

Analysis of the available literature has shown that the aspect of the application of physiotherapeutic techniques in the prevention of FI is a poorly researched issue, where only a few studies have focused on this topic. Nevertheless, bearing in mind the main components that determine stool continence, it is important to maintain the proper function and efficiency of PFM, including anal sphincters, in the prevention of FI [66]. For FI prevention in women, attention should be paid to the period of pregnancy and childbirth. Bearing in mind that childbirth is a significant risk factor for perineal injury and FI development, the possibility of implementing primary and secondary prevention of FI seem to be particularly important. Bø et al. [44] examined the influence of regular fitness training, including PFM exercises, in pregnant women for mitigating postpartum incontinence. Incorporation of PFMT into standard fitness training was not effective in relieving postpartum incontinence, which the authors explained by a lack of individual instruction regarding PFM contraction and no control of the contraction correctness by transvaginal palpation [44]. A similar study was conducted by Stafne et al. [45], where PFM exercises were added to the general exercise course consisting of aerobic exercise, stretching, and balance training. Additionally, pregnant women were obliged to perform training at home. They were encouraged to perform PFM contractions after a physiotherapist’s instruction and a transvaginal assessment of the correctness of PFM contraction. PFMT conducted in this way significantly reduced the intensity of both UI and FI incidents [45]. These studies emphasized the important role of professional instruction and transvaginal control of contraction in the effectiveness of PFMT. Detailed descriptions of these studies are presented in Table 4A. Physiotherapeutic prevention of FI includes maintaining proper tension and PFM strength. The basic procedure is PFM exercises, which can be supported by biofeedback [24,67]. In the case of PFM hypertonic tension, it is necessary to relax them by appropriate therapy [67].

Another physiotherapeutic technique in relation to which the literature confirms its effectiveness in the prevention of FI is perineal massage performed in late pregnancy. Antenatal perineal massage provides relaxation, improves the blood flow within the perineum, and makes the pelvic floor muscles more flexible [57]. Studies have shown that pelvic floor massage is safe and well tolerated by women [68] and may significantly reduce the risk of perineal injuries during delivery [69]. As perinatal perineal injuries are a risk factor for the development of FI [70], such massage may provide a preventive measure for maintaining proper continence [71]. Moreover, the consequences of perinatal injuries of the pelvic floor include not only the development of urinary or fecal incontinence but also perineal pain, sexual disorders, as well as decreased quality of delivery [69]. The study carried out by Ugwu et al. [57] showed that 10 min of daily massage from the 34th week of pregnancy reduces the risk of developing incontinence of feces, urine, and gases. However, Eogan et al. [65], in a similar study, observed only a tendency to reduce the risk of anal sphincter injuries could be caused by the shorter massage time (5 min). Therefore, it is recommended that it is performed daily for 10 min from the 34th week of pregnancy until delivery [57,72]. Antenatal perineal massage can also be combined successfully with other physiotherapeutic techniques, such as pelvic floor training [73]. Descriptions of the studies included in this review are presented in Table 5.

Referring to the issue of FI prevalence, the role played by women’s education and proper toilet habits cannot be ignored. Appropriate toilet habits are particularly important, both in the prevention and treatment of PFM dysfunction. The natural position for defecation is the tuck position, in which the anorectal angle becomes open and allowing the fecal mass to move freely. This position should be reached on the toilet by placing a support under the feet. This avoids pushing and, thus, increases pressure in the intra-abdominal cavity, lowering the PFM and weakening them [74]. The toilet should be used at the same time, as the nervous system is then accustomed to regularity. After using the toilet, the PFM should be always pulled up as during their exercise [2]. Proper diet and hydration should be also taken, in order to ensure the proper consistency of the stool [75]. Physical activity is also important here, which contributes to maintaining proper body weight and accelerating intestinal passage, thus avoiding constipation [13].

In conclusion, proper women’s education and correct toilet habits are important for the prevention of FI, as well as the implementation of PFM training by a specialist, which should be preceded by verification of the functional state of the pelvic floor, in order to adjust the training to the woman’s needs. In late pregnancy, perineal massage may be considered, in order to better prepare the structures for delivery and reduce the risk of perineal injuries.

## 5. Physiotherapeutic Techniques for the Treatment of Fecal Incontinence

Both surgical and conservative methods are used for the treatment of FI. Surgical interventions are aimed at anatomical and functional correction of the rectum, pelvic floor, or anal sphincters. In turn, conservative methods of FI treatment include physiotherapeutic techniques [76], such as patient education, pelvic floor and sphincter muscle training with or without biofeedback, electrostimulation, and manual therapy techniques [77]. Bearing in mind the recognition of physiotherapy as an important element in the therapy of women’s FI, below, we present a systematic review of the techniques described in the literature.

### 5.1. Pelvic Floor and Anal Muscle Training for the Treatment of Fecal Incontinence

The main objectives of physiotherapeutic muscle training in FI are to increase the strength, tension, endurance, and co-ordination of anal sphincter and pelvic floor muscles [18,77]. Anal training is based on the co-ordination of anal sphincters as well as isolation of their contraction [77]. An increase in the tension and strength of anal sphincters can lead to an improvement of anal canal capacity and facilitate the process of defecation [78,79], as well as potentially improving the level of resting anus closure pressure [80]. Therefore, for a better therapeutic effect and depending on the stage of FI advancement, muscle training can be combined with other techniques [77].

Exercise patterns in FI differ in type, number, and intensity of exercises from one exercise pattern for everyone (e.g., 10 strong contractions, per 5 s, five times a day), such that the training program should be individualized depending on the initial muscle parameters (i.e., strength and endurance) [80]. It is important that PFM training reduces the risk of developing FI, compared to standard care [45] or lack of exercise [46], and that it is more effective in the case of supervised training [45,46], as well as when preceded by transvaginal assessment and correction of PFM contraction [45]. Research suggests that early PFMT during the puerperium can effectively reduce the consequences of severe obstetric injuries [62]. In addition, to achieve a positive PFM training effect, the exercise should be done regularly [44]. It should also be noted that not every woman can perform the correct PFM contraction, in spite of instruction, which significantly reduces or even excludes the effectiveness of training. In such a situation, it is necessary to implement techniques aimed at PFM sensitization. It is estimated that nearly 25% without UI/FI symptoms and over 70% of women with PFM disorders face this problem. In these cases, basing physiotherapy solely on PFM exercises will be limited in effectiveness, as summarized in our previous work [81]. Therefore, PFM training should be preceded not only by a verbal exercise instruction, but also by transvaginal control and supervision by a physiotherapist in order to achieve optimal therapeutic effects. Unfortunately, most of the studies on the effectiveness of muscle training in the treatment of fecal incontinence have been carried out in the general population (both men and women) and it is often not possible to separate the results only for women. Moreover, it should be noted that PFMT is often the basic procedure recommended for control groups, in relation to which the effectiveness of tested techniques is measured. This proves the high recognition of exercise as a form of primary action in the treatment of FI. PFMT has been recommended by ICI-ICS as an effective method in the treatment of FI with a grade of recommendation of B [82,83]. In addition, ICI has recommended exercise as the first line of FI therapy [82]. Descriptions of the studies included in the review are presented in Table 4B.

### 5.2. PFM Training with Biofeedback for the Treatment of Fecal Incontinence

Inability to proper identify and isolate of PFM contractions have been included among the main obstacles to urogynoecological rehabilitation [79]. Biofeedback is one of the techniques that supports the learning and facilitation of PFM exercises [84], helping to isolate appropriate PFM [52] and anal sphincter contractions [24] without the co-contraction of other muscles. Biofeedback is a commonly used technique based on operant conditioning by using devices that provide the patient with acoustic or visual feedback on the quality of muscle activation. In the management of FI, biofeedback techniques include anorectal manometry and surface or endoanal EMG. Depending on the technique used, the patient receives information about their muscle activity or a change in anal canal/rectal pressure [85].

Biofeedback training improves rectal sensory function, strength, and the co-ordination of pelvic floor muscles [86] and, as research has shown, is effective in alleviating FI symptoms in nearly 75% of cases [61]. Improvement in the quality of life and/or a reduction in the severity of symptoms has been observed both in patients with idiopathic FI [47], with FI of different etiologies [61], and with fecal incontinence associated with scleroderma [60]. A positive effect of BF therapy has also been noted in patients after childbirth complicated by damage to the sphincters [52,58]. BF training has been demonstrated to be effective both in the model of pre- or post-FI surgery due, for example, to damage to the sphincters [50,51], as a stand-alone therapy in moderate FI [60], and in the prevention of FI [49]. BF can be combined with PFM training, FI education and pharmacological treatment, which effectively reduced FI symptoms and risk of FI development [47,48]. It should be noted that BF training can be successfully performed in both outpatient and home-based conditions [87], as well as in different positions and movements [88]. A significant difficulty in the physiotherapy of pelvic floor dysfunction is the low effectiveness of unsupervised home training, which has been particularly emphasized by research on PFM training [49]. Nevertheless, the protocol proposed by Damin et al. [59], testing a new BF device for home training, showed promising results in both the reduction of FI symptoms as well as a significant improvement in the quality of life of women. Moreover, studies have shown that, by combining training with biofeedback, a better therapeutic effect can be achieved in the management of FI than by education or PFM exercises alone [80]. The significant number of studies evaluating the effectiveness of this method in alleviating the symptoms of FI led it to being recommended by the ICI-ICS at the highest level (Level 1, grade A); however, it is recommended in the second line of therapy [83]. Nevertheless, it should be kept in mind that the necessary conditions for the application of the BF technique is the appropriate strength of the muscles, making it possible to contract them. In the case of patients with severe muscle weakness, it is appropriate to first apply electrical stimulation or PFM sensitization techniques. For this reason, prior to incorporation of this technique into FI therapy *per vaginam* or *per rectum*, PFM examination should be performed by an experienced physiotherapist. This allows for the assessment of whether the possible lack of a perceptible contraction of PFM is the result of significant muscle weakness, the inability to activate them, or the need to use PFM sensitization techniques. Descriptions of the studies included in the review are given in Table 6.

### 5.3. PFM Electrostimulation for the Treatment of Fecal Incontinence

Electrical stimulation is a technique used to treat many pelvic floor dysfunctions which passively contracts the stimulated muscles (here, PMF or anal sphincters). It improves the tension and muscle strength, as well as raises patient awareness about PFM contractions. To activate the reflex initiating peri-urethral sphincter, anal sphincter, or entire pelvic floor contraction, proper functioning of the spinal arc is essential [89]. Electrostimulation of the pelvic floor can be performed with surface perineal electrodes, as well as vaginal and rectal electrodes [90]. In endoanal electrical stimulation, the vulva nerve and anal sphincter are chronically stimulated [56], which leads to improved strength and endurance of the striated muscle (external anal sphincter) in FI patients [58]. Electrostimulation of the rectum reduces the tendency of the sphincter to fatigue and improves the sensory function of the rectum [87]. In most protocols, high-frequency stimulation at 50 Hz for 15–20 min twice daily is recommended [53,56,63]. Electrostimulation is often used in combination with other physiotherapeutic methods, which makes it difficult to separate the effects of these interventions [90]. Furthermore, electrostimulation is considered a non-invasive, low-cost, and easily accessible therapeutic method for the treatment of FI [91]. The effectiveness of electrostimulation has been demonstrated in women with FI after deliveries complicated with damage to the anal sphincters [53,54] and in women with chronic [55,56] or idiopathic FI [63]. The effectiveness of ES in alleviating FI symptoms has also been compared with other physiotherapeutic techniques such as BF [54] or PFMT [56]. The impact of ES on the quality of life has also been assessed [53,54,55].

It should be noted that electrostimulation is often combined with PFM training and biofeedback to identify contractions and increase PFM strength [92]. As a method of passive muscle stimulation, it is a valuable therapeutic tool in the case of severe muscle weakness, in which the contraction necessary for PFM or BF training is ineffective. PFM electrostimulation has been recommended by ICI-ICS as an FI treatment with the grade of recommendation of B. Similarly to BF, its use is recommended in the second line of treatment [83]. Descriptions of the studies included in the review are given in Table 7.

### 5.4. PFM Magnetic Stimulation for the Treatment of Fecal Incontinence

Magnetic stimulation (MS) is a non-invasive method that has been suggested for use as an alternative to electrostimulation [93]. In MS, an electric current is induced by a time-varying magnetic field [94]. The key to the effectiveness of MS in the treatment of pelvic floor disorders is depolarization of nerve fibers, which leads to a gradual increase in strength and endurance of the PFM [95]. Due to the lack of an internal probe and the ability of the magnetic field to pass through clothing, MS is painless and well-tolerated by patients with pelvic floor dysfunction [94,96]. Nevertheless, this method has been verified in only a few studies, which makes it impossible to recognize it as an effective and recommended technique in the treatment of FI at present. Further research is still needed; however, the existing results are promising. In the study by Brusciano et al. [95], a frequency of 50–60 Hz was used in the treatment of FI in both women and men. The treatment time was 15 min, performed once a week for 8 weeks [95]. In turn, Schobeiri et al. [64] verified the efficacy of MS in FI treatment exclusively among women. The frequency of the magnetic field was increased gradually from 5 to 50 Hz. A single therapeutic session lasted 20 min and was performed twice a week for two months. Both studies showed a significant decrease in symptoms of FI [64,95]. Nevertheless, this technique has not been recommended by the ICI-ICS, so far. It should also be mentioned that MS can cause minor side effects [64]. At 100% magnetic field intensity, patients experienced tingling and numbness in the buttocks and back of the thigh. Moreover, after the end of stimulation, most of the examined women felt dull fatigue in the buttocks [64]. Detailed descriptions of the studies included in the review are given in Table 8.

### 5.5. Reccomendations for Conservative FI Treatment

According to the recommendations of the International Continence Society (ICS) from 2019 for the treatment of women with fecal incontinence, education (grade of recommendation (GoR) B/C), dietary change (GoR B), supplements (GoR A), change of toilet habits (GoR C), and the use of absorptive products (GoR B) are recommended as a primary intervention [82], while PFMT (GoR B) and Biofeedback (GoR A) are recommended as secondary interventions in the treatment of FI [82]. Nevertheless, according to the recommendations of the 6th edition of International Consultation on Incontinence (ICI), PFMT should be implemented as an early intervention in FI treatment (GoR B) [83], while BF should be the second line after behavioral methods and conservative treatment (GoR A). Moreover, studies have confirmed that BF is more effective in combination with PFMT (level of evidence 1). Stand-alone PFMT appears to be effective, but the results so far are contradictory (level of evidence 2). Home BF or ES is an effective form of therapy, but younger patients benefit more than older patients (level of evidence 2, grade of recommendation B). In the 6th edition of the ICI, the opinion on ES was also changed. Studies have suggested that low ES frequencies are not effective, even when combined with BF, but ES therapy at 30 Hz with BF performed twice a day for at least six months is effective (level of evidence 2, grade of recommendation B). At present, MS therapy is not recommended by the ICI for the treatment of FI [82,83]. The quality of evidence of physiotherapy techniques in FI is presented in Table 9 (according the ICI-ICS standards).

## 6. Limitation of the Study

The presented review has some limitations, including the small number of papers describing the effects of physiotherapy in the treatment of FI in women. We had to reject many papers, due to the fact that they were related to both women and men and as it was not possible to separate the results by gender. Moreover, the presented studies recruited a relatively small number of patients who showed a large variety of FI symptoms (from mild to severe), which were often not included in the analysis of the effectiveness of a given method. The small number of papers, as well as the low quality of many of them, made it impossible to carry out a statistical evaluation of the effectiveness of the methods and meta-analysis. However, it should be noted that the review was not limited to only RCTs. In addition, works from the years 2000–2020 were analyzed, which is a wide range of time covering the current literature.

## 7. Conclusions

The possibility of fecal incontinence in women is generally underestimated by doctors, even though it affects about 0.25–6% [3] of the population. Some of the most common causes of these problems are changes resulting from iatrogenic trauma in young women, systemic diseases, and inadequate hygiene habits, which lead to symptoms at a later age. Some of the causes of these complaints can be eliminated by effective prophylaxis, both in the form of education introducing appropriate toilet habits (to reduce intra-abdominal pressure) and dietary habits (to maintain proper consistency of stool), early diagnosis of systemic diseases occurring with polyneuropathy, and active prevention through physiotherapy techniques. In each of these cases, there should be close co-operation between the patient and the therapist. It seems that a good solution is to create teams consisting of a doctor, a physiotherapist, a dietician, and a psychologist. Therefore, FI management should be multidisciplinary. In the case of ailments, correct diagnosis based on correct subjective and physical examination (including palpation) is essential. It is also extremely important to assess the defects in the patient’s posture. Treatment, especially in cases with a lower degree of severity of the disease, should start from conservative management including physiotherapy and pharmacotherapy.

On the basis of the presented results, it can be concluded that physiotherapy is an effective method of conservative FI treatment in women. Physiotherapeutic techniques in FI management include pelvic floor muscle training (mainly anal sphincter), BF, ES, MS, and perineal massage. Physiotherapy plays an important role in the treatment of pre- and post-operative FI. PFM training can also be performed by pregnant women, in order to reduce the risk of FI development in the antenatal and postnatal periods. Moreover, Lacima et al. showed a tendency for younger patients to obtain better clinical results after treatment than older patients [61].

Regular and appropriately adjusted rehabilitation leads to increases in strength, endurance, tension, and co-ordination and sensory activity of anal sphincter muscles and levator ani. In order to achieve the best possible results in FI physiotherapy, the combination of different techniques is recommended. In patients who have problems performing conscious, isolated contraction of the anal sphincter muscles, PFM training in combination with BF should be conducted. In the latest recommendations issued by the ICS (2019), the highest level of recommendation (A) was achieved by BF. In turn, PFMT and ES were classified at level B. Additionally, magnetic stimulation and perineal massage can be used in the treatment of FI. Physiotherapeutic techniques demonstrated high efficiency in reducing the symptoms of FI in many studies. However, due to the small number of high-quality publications on the use of physiotherapy in the treatment of FI, RCT studies are still needed.

## Figures and Tables

**Table 1:** PERFECT examination scheme of pelvic floor muscle (PFM).

P	Performance	Strength of maximum voluntary contraction (MVC) evaluated in the Oxford Modified Scale	
		0	no contraction
		1	flickering or pulsation
		2	poor tension without lifting the vaginal walls
		3	moderate tension with vaginal walls lifting without resistance
		4	contraction with lifting of vaginal walls leading to join fingers in the vagina without therapist resistance
		5	strong contraction leading to join fingers in the vagina against resistance
E	Endurance	Muscle strength assessed in seconds (0–10) as the ability to maintain an MVC until it falls to 50% of MVC	
R	Repetition	Number of MVC repetitions (0–10) of the length diagnosed in the endurance section	
F	Fast	Performance of fast twitch fibers evaluated as the number of repetitions (0–10) of one-second MVC	
E	Elevation	Cefalo-ventral elevation of PFM	
C	Co-contraction	Reflex co-contraction of the transverse abdominal muscle during the contraction of the PFM	
T	Timing	Involuntary PFM contraction during coughing provocation	

**Table 2:** Evaluation of the quality of research in Pedro scale included only RCT studies [43].

Study	EC *	RA	CA	BC	BS	BT	BA	AF	ITA	BGC	PEaV	TS
Bø, 2011 [44]	+	+	+	+	-	-	-	-	-	+	+	5
Stafne, 2012 [45]	+	+	+	+	-	-	-	+	+	+	+	7
Johannessen, 2017 [46]	+	+	-	+	-	-	-	-	+	+	+	5
Ilnyckyj, 2005 [47]	+	+	-	-	-	-	-	-	-	+	-	2
Sjödahl, 2015 [48]	+	+	+	+	-	-	-	+	-	+	+	6
Peirce, 2013 [49]	+	+	+	-	-	-	-	+	-	+	-	4
Davis, 2004 [50]	+	+	-	+	-	-	-	-	-	+	+	4
Ghahramani, 2016 [51]	+	+	+	+	+	+	+	+	+	+	+	10
Sigurdardottir, 2020 [52]	+	+	-	+	-	-	+	+	-	+	+	6
Naimy, 2007 [53]	+	+	-	-	-	-	-	-	-	+	+	3
Mahony, 2004 [54]	+	+	+	+	-	-	+	+	-	+	+	7
Healy, 2006 [55]	-	+	-	-	-	-	-	-	-	+	+	3
Cohen-Zubary, 2015 [56]	+	+	+	+	-	-	-	-	+	+	+	6
Ugwu, 2018 [57]	+	+	+	+	-	-	-	+	-	+	+	6

* Eligibility criteria it is not included in the total score; EC, Eligibility criteria; RA, Random allocation; CA, Concealed allocation; BC, Baseline comparability; BS, Blind subjects; BT, Blind therapists; BA, Blind assessors; AF, Adequate follow-up; ITA, Intention-to-treat analysis; BGC, Between-group comparisons; PeaV, Point estimates and variability; TS, Total score (1 point: “+”; 0 point: “-”).

**Table 3:** Characteristics of comparators of studies qualified for the review.

Study	Qol	Depression	UI	FI	CI	SPF	PFMF	Other
Bø, 2011 [44]			+	+				GI
Stafne, 2012 [45]			+	+			+	TD
Johannessen, 2017 [46]				+	+		+	EaU
Ilnyckyj, 2005 [47]				+			+	BD
Sjödahl, 2015 [48]				+			+	BD, EaU
Peirce, 2013 [49]	+	+	+	+	+		+	EaU
Coffey, 2002 [58]				+		+	+	
Damin, 2017 [59]	+			+			+	
Collins, 2016 [60]	+			+			+	
Davis, 2004 [50]	+			+		+	+	EaU
Ghahramani, 2016 [51]				+			+	EaU
Lacima, 2016 [61]				+	+		+	EaU, PNTML, BD
Sigurdardottir, 2020 [52]			+	+	+		+	
Mathé, 2016 [62]	+		+	+	+			GI
Naimy, 2007 [53]	+			+	+			
Worsøe, 2011 [63]	+	+		+			+	BD
Mahony, 2004 [54]	+			+			+	EaU
Healy, 2006 [55]	+			+				EaU, PNTML
Cohen-Zubary, 2015 [56]		+	+	+			+	GI
Shobeiri, 2007 [64]			+	+			+	EvU
Ugwu, 2018 [57]			+	+	+			GI
Eogan, 2006 [65]				+	+			EaU, PP

Qol, Quality of life; UI, Urinary Incontinence; FI, Fecal Incontinence; CI, Childbirth injury; SPF, Self-perceived function; PFMF, Pelvic Floor Muscle Function; GI, gas incontinence; EaU, endoanal ultrasound; EvU, endovaginal ultrasound; PNTML, pudendal nerve terminal motor latency; BD, Bowel symptoms diary; TD, training diary; PP, perineal pain.

**Table 4:** Characterization of selected studies on the effects of pelvic floor muscle training (PFMT) on the fecal incontinence prevention (A) and treatment (B).

Reference	Main Objective	Participants	Intervention	Outcome
(A) Pelvic floor muscle training (PFMT) in fecal incontinence prevention				
Bø et al. (2011) Norway [44] A randomized controlled trial study	The effectiveness of PFM exercises conducted during general fitness classes in pregnant women for mitigating postpartum incontinence.	84 pregnant women Exp.: 42 (aged 31.2 ± 3.7 years) Con.: 42 (aged 30.3 ± 4.4 years)	Exp.: 1 h general fitness classes with PMFT (3 × 8–12 PFM contractions, hold 6–8 s); 2–3 times a week per 12 weeks + 30 min additional home exercises Con.: w/o interventionAssessment: SI, ICIQ–UI SH,	No efficacy of PFMT conducted during general fitness classes in reduction of FI symptoms assessed 6–8 weeks after childbirth.
Stafne (2012) Norway [45] A randomized controlled trial study	Comparison of the effectiveness of general exercise course including PFMT with standard care in the prevention of UI and FI in late pregnancy	761 pregnant women Exp.: 396 (aged 30.5 ± 4.4 years) Con.: 365 (aged 30.4 ± 4.3 years)	Exp.: general exercise with PFMT (1 h, once a week under physiotherapist supervision + 45 min at home, twice a week per 12 weeks) Con.: standard care w/o exercise Assessment: SI, St. M.S, questionnaire about PFMT	Pregnant women who regularly participated in the training program reported UI and FI less frequently in late pregnancy.
(B) Pelvic floor muscle training (PFMT) in fecal incontinence treatment				
Johannessen (2017) Norway [46] A randomized controlled trial study	Assessment of the effectiveness of individualized PFM training in the treatment of FI in the postpartum period	109 women with FI 1 year postpartum Exp.: 54 (aged 29.7 ± 4.3 years) Con.: 55 (aged 30.6 ± 3.8 years)	Exp.: individually adapted home PFMT program: 3 sets of 8–10 maximum PFM contractions per day, 3 s long with progression to 10 or 12 s with 3 fast contraction at the end, for 6 months Con.: written information about PFMT, training was not obligatory. Assessment: St. M.S, EaU, manometry, VPFMC	Both interventions reduced the symptoms of FI, howeverregular, individualized PFMT in the postpartum period reduced them significantly more, which has been described as a clinically significant effect.
Mathé et al. (2016) France [62] A retrospective observational study	Comparison of the effectiveness of early PFMT and/or standard rehabilitation for FI symptoms after vaginal deliveries complicated by ≥3rd degree of perineal tears	167 women with ≥3rd degree of perineal tears after vaginal delivery Con.: 83 (age: 29.5 ± 4.7 years) Exp.: 84 (age: 30.6 ± 4.1 years)	Con.: standard rehabilitation, PFMT from 6–8 weeks of puerperium + BF as supports + education Exp.: early rehabilitation, PFMT after 30 days postpartum (6–10 series of PFMT twice a day) + standard rehabilitation (as in the group Con.) Assessment: Modified version of the Jorge and Wexner questionnaire	The implementation of early rehabilitation significantly reduces FI, GI, and UI in women after childbirth complicated by massive perineal damage, this result was significantly better than that obtained after standard rehabilitation.

Exp., experimental group; Con., control group; PFMT, pelvic floor muscle training; FI, fecal incontinence; UI, urinary incontinence; GI, gas incontinence; PFM, pelvic floor muscles; SI, severity index; ICIQ-UI SH, International Consultation of Incontinence Questionnaire Urinary Incontinence Short Form; St. M.S, St. Mark’s score; VPFMC, voluntary pelvic floor muscle contractions; EaU, endoanal ultrasound; BF, biofeedback.

**Table 5:** Studies assessing the role of antenatal perineal massage in fecal incontinence prevention.

Reference	Main Objective	Participants	Intervention	Outcome
Ugwu et al. (2018) Nigeria [57] A randomized controlled trial study	Evaluation of the effectiveness of APM in the prevention of perineal injuries and FI development.	108 primiparous at 34–36 weeks of pregnancy MG: 53 (Average Age: 28.02 Years) Con.: 55 (Average Age: 28.77 Years)	MG: perineal massage, 10 min a day until delivery Con.: no intervention Follow up after 12 weeks Assessment: Diary of APM	APM reduces the frequency of incisions and other perineal injuries. Moreover, it lowers the risk of FI after childbirth.
Eogan et al. (2006) [65] A prospective observational study	Assessment of the impact of APM on the prevention of stool disorders	MG: 100 primiparous at 34 weeks of pregnancy (Average Age: 30.00 Years) Con.: 79 (Average Age: 25.9 Years)	MG: perineal massage, 5 min a day until delivery Con.: no intervention Follow up after 3 days and 3 months of postpartum Assessment: PS, Continence Score, Anal manometry, EaU	APM reduces postpartum perineal pain. There were no significant differences in the manometry results between the two groups.

FI, fecal incontinence; APM, antenatal perineal massage; MG, massage group; Con., control group; PS, Pain Scale; EaU, endoanal ultrasound.

**Table 6:** Characterization of selected studies on the effects of the biofeedback (BF) technique on the severity of fecal incontinence symptoms.

Reference	Main Objective	Participants	Intervention	Outcome
Ilnyckyj et al. (2005) Canada, [47] A randomized controlled trial study	Assessing whether BF has any specific effect beyond the standard educational intervention	18 women with FI history ≥ 6 months (aged 26–75 years) Con.: 11 Exp.: 7	Con.: education on FI and PFM and verbal instruction of PFM exercises (6 times a day, 5 contractions with maximum possible force and holding time separated by a 20 s pause). Exp.: education and PFM exercise as in the Con.: group + BF training Assessment: manometry, diary	Education with exercise instruction and BF effectively minimize the symptoms of FI in women. In both groups, there was a significant improvement in squeeze duration; however, resting and squeeze pressures improved only in the group with BF.
Sjödahl et al. (2015), Sweden [48] A randomized controlled trial study	Evaluation of the therapeutic effect of BF alone or in combination with pharmacotherapy in the treatment of FI.	57 women with FI history ≥ 1 episode of FI within 2 weeks BF + Pharmaco: 29 (median age 62 years) Pharmaco + BF: 28 (median age 57 years)	A combination of BF therapy and subsequent pharmacotherapy or vice versa BF: education, behavioral instructions and surface electro-BF training with anal plug (1–6 session during 4–6 months with individual home exercise program) Pharmaco: loperamide and stool-bulking agents for 2 months Assessment: 14 day symptoms diary, 3D-EAUS, anal function	Both type of combined interventions significantly reduced the FI symptoms and was more effectiveness than BF or pharmacotherapy alone (significant reduction in urgency, number of loose stools, leakages without forewarning, and passive leakages were observed).
Peirce et al. (2013), Ireland [49] A randomized controlled trial study	Comparison of early home BF physiotherapy with PFM exercises in the initial management of women sustaining a primary third-degree perineal tear.	120 Primiparous women with a primary third-degree perineal tear Exp.: 30 (aged nd) Con.: 90 (aged nd)	Exp.: home EMG BF therapy with the intra-anal probe, twice a day for 3 months (10 contractions, 5 s duration, 10 s rest) Con.: home PFMT (5 min standard Kegel exercise) twice a day for 3 months. Assessment: anorectal manometry, EAU, CCCS, RFI QoL	No additional benefits of BF therapy compared to PFMT have been demonstrated. There was no difference in the manometric test, FI symptoms and quality of life between the groups.
Coffey et al. (2002), USA [58] Case Report	Evaluation of the effectiveness of multifactorial therapy designed to reduce the PFM dysfunction and FI symptoms.	30 year old woman with FI symptoms from 8 years which started after the first delivery (vacuum extraction, perineal incision, child’s weight 4.16 kg)	Education, EMG BF, strengthening exercises, PFM relaxation training, soft tissue techniques, Follow up: 4 months Assessment: authors questionnaire, digital palpation, EMG BF	As a result of the therapy improvements have been achieved in such aspects as the quality of life, PFM strength, endurance and control. FI symptoms was also improved.
Damin et al. (2017), Brazil [59] A pilot study	Verification of the usefulness of a novel portable biofeedback device in the FI treatment	10 women with FI, without any previous treatment (aged 50–73 years)	BF training: daily for 28 days, 20 min in three phases (1) 5 series of 10 2-s contractions with a 2-s rest between contractions, (2) 2-min break, (3) 5 series of 10 contractions during 5 s with a 5 -second rest. PFM contractions of maximum strength Assessment: Wexner scale, FIQL	A reduction in FI symptoms and an increase in QoL were achieved. The BF device allows for effective training for FI treatment at home.
Collins et al. (2016), Australia [60] A case-control study	Assessment of the effectiveness of anorectal BF in the treatment of FI and QoL improvement among patients with scleroderma compared to patients with functional FI.	39 women with FI G.I.: 13 women with FI and scleroderma (median age 59, IQR 47–65 years) G.II.: 26 women with functional FI (age- and parity-matched)	Both groups: supervised BF training (30–60 min), once a week for 6 weeks, education, BF and PFMT learning, anal sensory training. Follow up: 6 weeks and 6 months after the end of therapy Assessment: FISI, manometry	BF training improved quality of life and stool control in both groups. Patients with scleroderma benefit from BF comparable as patients with functional FI.
Davis et al. (2004), United Kingdom [50] A randomized controlled trial study	To evaluate the effect of BF as adjuvant therapy in women after anal sphincter surgery.	31 women with FI referred to a secondary/tertiary colorectal unit Con.: 17 (aged 60.29 ± 13.59 years) Exp.: 14 (aged: 60.71 ±10.04 years)	Con.: anal sphincter surgery Exp.: BF 3 months after sphincter surgery; session duration 1 h, once a week for 6 weeks, education, BF, and PFMT twice a day at home. Assessment: CGSS, manometry, EaU	Anal sphincter surgery significantly reduces the FI symptoms. Postoperative BF therapy improves the long-term QoL of patients.
Ghahramani et al. (2016), Iran [51] A randomized controlled trial study	Assessment of the effect of BF applied before and/or after surgery on FI symptoms in women with sphincter damage.	27 women with the anal sphincters damage during childbirth Exp1: 9 (aged: 41.1 ± 13.12 years), Exp2: 9 (aged: 36.8 ± 16.45 years), Con.: 9 (aged: 44.85 ± 15.32 years),	Exp1: BF 3 months before and 6 months after surgery Exp2: BF 6 months after surgery Con.: operation only BF: supervised training—10 sphincter muscle contractions maintained for 5 s, education; at home—100 contractions of the anal sphincter muscles twice a day Assessment: The Wexner scale, manometers, EaU	Surgery alone and in combination with BF reduce the FI symptoms in women. The use of BF before and/or after surgery provides better FI improvement than surgery alone (Wexner scale), but not in manometry.
Lacima et al. (2016), Spain [61] Observational study	Prospective identification of clinical factors that can predict the efficacy of BF for FI treatment and evaluate the utility of tests in predicting outcomes of treatment.	135 women with fecal incontinence of varying etiology (aged: 60.6 ± 11.5 years)	A minimum of 4 BF sessions + anal sphincter exercises at home; 10 min, twice a day Assessment: manometry, rectal sensory testing, EaU, PNTML, questionnaire about symptoms	BF therapy was effective in the treatment of FI. Clinical factors and tests to predict treatment outcomes could not be described
Sigurdardottir et al. (2020), Iceland [52] A randomized controlled trial study	Assessment of the effects of PFMT with BF facilitation in the early postpartum period on UI and FI symptoms and related problems, as well as the strength and endurance of PFM.	84 women with UI and FI symptoms Exp.: 41 (mean age: 28 ± 4.3 years) Con.: 43 (mean age: 29 ± 5.3 years)	Exp.: supervised PFMT with vaginal EMG-BF to facilitate—once a week for 12 weeks, session duration 45–60 min; home PFMT 3 x 10 PFM contractions. Training started in the 9th week post-partum Con.: no intervention Assessment: APFQ, manometry	PFMT with BF facilitation increased the strength and endurance of both PFM and anal sphincter, but the frequency of UI and FI after 6 and 12 months remained unchanged.

Exp., experimental group; Con., control group; G., group; FI, fecal incontinence; BF, biofeedback; QoL, quality of life; PFM, Pelvic Floor Muscle; PFMT, pelvic floor muscle training; SCI, Spinal cord injury; EAU, endoanal ultrasonography; CCCS, Cleveland Clinic Continence Score; RFI QoL, Rockwood Faecal Incontinence Quality of Life; FIQL, Fecal Incontinence Quality of Life Scale; CGSS, Continence Grading Scale Score; FISI, Fecal incontinence severity index; UI, urinary incontinence; PNTML, pudendal nerve terminal motor latency; APFQ, Australian Pelvic Floor Questionnaire; 3D-EAUS, three dimensional endoanal ultrasonography; EMG-BF, electromyographic biofeedback.

**Table 7:** Characterization of selected studies on the effects of electrostimulation (ES) on the severity of fecal incontinence symptoms.

Reference	Main Objective	Participants	Intervention	Outcome
Naimy et al. (2007), Norway [53] A randomized controlled trial study	Comparison of the effect of BF vs. ES in the treatment of postdelivery FI.	49 women with FI (≥3rd degree of perineal tears) (aged 22–44 years) BF: 24 ES: 25	2 sessions with a therapist, and then twice a day for 8 weeks at home. BF: 30 min, 5 sets of 3 s, 10 s and as long as it can be kept with a minimum 50 percent of amplitude of the three-second contraction ES: with anal probe; frequency 30–40 Hz, pulse width: 200 ms, up to 80 mAmp, time: 20 min. Assessment: Wexner score, FIQL, RQL	No improvement was observed after BF or ES therapy in FI symptoms (Wexner score) and quality of life (FIQL) in women with postdelivery FI problem. Both therapies improved the subjective perception of fecal incontinence control by patients.
Worsøe (2011), Denmark [63] A prospective descriptive study	Assessment of the effects of DGN stimulation on FI symptoms.	Nine women with idiopathic FI (median age, 60 years; 34–68 years)	ES: twice a day for 3 weeks; 15 min, pulse width 200 μs; frequency 20 Hz. Assessment: VAS, FIQL, bowel habit diary, EA USG, Wexner score, St. Mark’s scale, manometry	After electrostimulation of DGN, the symptoms of FI were reduced (Wexner score, St. Mark’s scale), and the effect was maintained also 3 weeks after the end of treatment.
Mahony et al. (2004), Ireland [54] A randomized controlled trial study	Comparison of the effectiveness of intra-anal EMG-BF with intra-anal EMG-BF combined with anal sphincter ES in the treatment of postpartum FI, as well as QoL of treated patients.	54 women with FI after obstetric injury BF + ES: 28 (median age 35 years, range 23–39), BF: 26 (median age 32 years, range 22–42)	In both groups: daily PFMT for 12 weeks BF + ES: PFEs with EMG-BF or intra-anal BF and intra-anal electrical stimulation for 20 min, once per week. BF: PFEs with EMG-BF or intra-anal BF once per week. Assessment: FIQL, questionnaire to determine continence score, manometry, EA USG	In both groups, there was significant improvement in FI symptoms and quality of life. ES did not bring any additional benefits.
Healy et al. (2006), Ireland [55] A prospective study	Comparison of the effectiveness of home and hospital therapy using low-frequency endoanal electrostimulation in alleviating FI symptoms.	38 women with FI (mean age: 55 years; range 40–78) G.I: 21 G.II: 17	G.I: low-frequency endo-anal ES at home with sequence of 3, 10, 20, 30, 40, 10 Hz frequencies (4 s on/4 s off), 1 h daily for 3 months G.II: low frequency endo-anal ES (15 min 10 Hz + 15 min 40 Hz) with biofeedback; 2 series 15 min; once a week under the supervision of a physiotherapist for 3 months Assessment: manometry, Wexner score, QoL	Low-frequency ES significantly reduced the symptoms of FI and improved quality of life. Daily use of home ES significantly improved rectal pressure (resting and squeeze pressure).
Cohen-Zubary et al. (2015) Israel [56] A randomized controlled trial study	Comparison of the effectiveness and costs of ES at home with BF training in women with FI.	36 women with chronic FI (mean age: 67.45 ± 7.2 years) ES: 18 (mean age: 66.6 ± 6.6) BF + PFMT: 18 (mean age: 68.3 ± 6.9)	ES: stimulation twice daily (25 min) for 6 weeks BF + PFMT: once a week supervised PFMT with BF (30–45 min) for 6 weeks and PFMT at home twice a day, 3 series, 10 contractions for 10 s. Assessment: VAS, VIS, HADS, intra-anal surface EMG	In both groups there was an increase in muscle strength as well as a decrease in FI symptoms. There were no adverse side effects.

G., group; FI, fecal incontinence; FIQL, fecal incontinence quality of life; RQL, reduced quality of life (on visual analog scale of 0 to 10); QoL, quality of life; DGN, dorsal genital nerve; EA USG, endoanal ultrasonography; ES, electrical stimulation; BF, biofeedback; VIS, Vaizey incontinence score; VAS, visual analog scale; HADS, Hospital Anxiety and Depression Scale; PFMT, pelvic floor muscle training; EMG-BF, electromyographic biofeedback; PFEs, pelvic floor electrostimulation.

**Table 8:** Characterization of the effects of magnetic stimulation (MS) on the severity of fecal incontinence symptoms in women.

Reference	Main Objective	Participants	Intervention	Outcome
Shobeiri et al. (2007), USA [64] A prospective cohort pilot study	Assessment of the usefulness of EXMI in alleviating FI symptoms in women with underactive pelvic floor.	16 women with FI and underactive PFM (mean age 57 years)	EXMI in a sitting position on a chair inducing an alternating magnetic field, frequency from 5 to 50 Hz, pulse 8 s, rest 4 s, for 20 min, twice a week for 8 weeks. Follow-up after 12 weeks Assessment: CCFIS, MR, endovaginal ultrasound, PFM examination with a Kegel Perineometer	EXMI alleviates the FI symptoms among women with an underactive pelvic floor (CCFIS). Pelvic floor rest and squeeze pressures have improved significantly.

EXMI, extracorporeal magnetic stimulation; FI, fecal incontinence; CCFIS, Cleveland Clinic Fecal Incontinence Score; MR, magnetic resonance; PFM, pelvic floor muscles.

**Table 9:** Quality of evidence of physiotherapy techniques in FI in the ICI-ICS standards.

Method	Level of Evidence	Grade of Recommendation	Line of Treatment (ICI)
PFMT	2	B [82]	Primary
BF	1	A [82]	Secondary
ES	2	B [83]	Secondary
MS	-	-	-

PFMT, Pelvic Floor Muscle Training; BF, Biofeedback; ES, Electrostimulation; MS, Magnetostimulation.
